# Diagnostic accuracy of dental caries detection using ensemble techniques in deep learning with intraoral camera images

**DOI:** 10.1371/journal.pone.0310004

**Published:** 2024-09-06

**Authors:** Sohee Kang, Byungeun Shon, Eun Young Park, Sungmoon Jeong, Eun-Kyong Kim

**Affiliations:** 1 Department of Dentistry, College of Medicine, Yeungnam University, Daegu, South Korea; 2 Research Center for Artificial Intelligence in Medicine, Kyungpook National University Hospital, Daegu, South Korea; 3 Department of Medical Informatics, School of Medicine, Kyungpook National University, Daegu, South Korea; 4 Department of Dental Hygiene, College of Science and Technology, Kyungpook National University, Sangju, South Korea; Danube Private University, AUSTRIA

## Abstract

Camera image-based deep learning (DL) techniques have achieved promising results in dental caries screening. To apply the intraoral camera image-based DL technique for dental caries detection and assess its diagnostic performance, we employed the ensemble technique in the image classification task. 2,682 intraoral camera images were used as the dataset for image classification according to dental caries presence and caries-lesion localization using DL models such as ResNet-50, Inception-v3, Inception-ResNet-v2, and Faster R-convolutional neural network according to diagnostic study design. 534 participants whose mean age [SD] was 47.67 [±13.94] years were enrolled. The dataset was divided into training (56.0%), validation (14.0%), and test subset (30.0%) annotated by one experienced dentist as a reference standard about dental caries detection and lesion location. The confusion matrix, area under the receiver operating characteristic curve (AUROC), and average precision (AP) were evaluated for performance analysis. In the end-to-end dental caries image classification, the ensemble DL models had consistently improved performance, in which as the best results, the ensemble model of Inception-ResNet-v2 achieved 0.94 of AUROC and 0.97 of AP. On the other hand, the explainable model achieved 0.91 of AUROC and 0.96 of AP after the ensemble application. For dental caries classification using intraoral camera images, the application of ensemble techniques exhibited consistently improved performance regardless of the DL models. Furthermore, the trial to create an explainable DL model based on carious lesion detection yielded favorable results.

## 1. Introduction

Dental caries is a disease that is common worldwide [1]. If it can be detected early, minimally invasive treatment is possible, which can contribute to tooth substance preservation more conservatively and effectively [2]. For example, in case of proximal surface caries, the resin infiltration technique was reported to be effective in preserving tooth substance of both marginal ridge and proximal contact by itself or along with internal tunnel restoration [3–5]. Therefore, this technique was recommended as a minimally invasive treatment by preempting surgical intervention among some non-cavitated caries [3]. An effective screening method to achieve a quick and exact diagnosis of dental caries is useful for both the patients and the dentist. For this reason, an intraoral camera, which can show enlarged images of the tooth surface with a high resolution on a computer monitor, is commonly used along with radiographs at dental hospitals in Korea.

The use of a convolutional neural network (CNN), a deep learning (DL) algorithm, is a very efficient method for image data processing [6–8]. With the application of CNN, the development of medical decision support systems has become a topic of interest in both the medical academia and industry [9]. In dentistry, there have been attempts to detect dental caries by using CNN models with various types of dental images [10–18], some of which were used to classify or localize dental caries lesions with dental X-ray images [10–18]. In other studies, dental caries lesions were classified using near-infrared light transmission illumination images [19, 20], or optical coherence tomography [21]. Periapical tooth lesions were also detected using images obtained by cone-beam computed tomography scans [22]. However, photographic images captured by an intraoral camera or smartphone having the advantage of convenience and safety are currently used for the application of an artificial intelligence (AI) model to screen dental caries in many studies [23–28] and demonstrated significant improvements in performance with various techniques [29–31]. For example, a previous study reported an accuracy of 0.81 and an area under the receiver operating characteristic curve (AUROC) of 0.84 using tooth surface segmentation in intraoral images [30].

Along with segmentation, ensemble techniques offer a way to enhance the performance of DL models by combining multiple models, each with its own strengths and weaknesses [32–34]. As this approach creates a more robust and accurate model, while also mitigating errors, improving generalization performance, and reducing overfitting, an ensemble model has been increasingly used in disease classification [35–37]. Thus, it is necessary to use an ensemble technique with intraoral camera images for dental caries classification for better performance.

Furthermore, creating DL models with explanation ability as well as better performance is important in medical fields. Due to the black-box nature of AI algorithms for classifying medical images, doctors may be reluctant to their clinical use [38]. To realize a more evidence-based diagnosis model, object detection can be used, which is a type of computer vision technique that estimates the position of specific objects in images or videos [39–41]. In the case of classification, it can be challenging for humans to understand the underlying mechanism of decision for classification of AI. However, by using object detection, the suspicious disease areas can be highlighted and indicated by bounding boxes, providing explainable evidence for the classification of the whole image.

Therefore, this study evaluated the diagnostic performance of some models, including ResNet-50, Inception-v3, and Inception-ResNet-v2, according to the application ensemble technique in intraoral camera image-based dental caries detection using end-to-end image classification. In addition, for an explainable detection of dental caries, image classification was done based on the object detection result using Faster R-CNN, of which diagnostic performance was evaluated according to ensemble technique. Through this evaluation, we aimed to examine the null hypothesis that an ensemble application in AI models could not improve overall performance in dental caries detection tasks.

## 2. Material and methods

### 2.1 Study protocol

A total of 540 patients who visited the dental clinic of the high-level general hospital at the metropolitan area in Korea were recruited from January 2021 to May 2022. Inclusion criteria for study participation were having favorable general health without infectious disease with at least one permanent tooth. However, those who could not cooperate to be photographed in the oral cavity because of cognitive or physical conditions or refused to provide written informed consent after receiving an explanation of the purpose and process of the study were excluded. Finally, 534 patients (male:285, female:249) whose mean [SD] age was 47.67 [±13.94] years were enrolled, and 2682 intraoral photographic images were included as a dataset, in which there were 3685 dental caries lesions (ICDAS code 4: 3273, ICDAS code 5 or 6: 412) in 1605 images. 491 participants had dental caries (ICDAS code 4: 287, ICDAS code 5 or 6: 204) among 534 patients. Just before capturing intraoral camera images, the target teeth were prepared by removing food debris and drying using a three-way syringe for 5 seconds with a saliva ejector without plaque or tartar removal. However, during taking a photograph, additional drying of teeth or isolation from saliva were not done. To collect various types of teeth images, no inclusion criteria were implemented. Therefore, dental restoration, non-carious tooth defects including stain or tooth wear, and saliva might be included in these images. Also, more than one tooth might be captured in one image. All images with a resolution of 1280 × 720 pixels were captured by an intraoral camera (Qraypen; AIOBIO, Seoul, Republic of Korea). The study procedure was approved by the Institutional Review Board of Kyungpook National University (KNU-2021-0097). Diagnostic/prognostic study design was applied to this study in accordance with the Declaration of Helsinki and the guidelines of the Standards for Reporting of Diagnostic Accuracy Studies (STARD) [42].

### 2.2 Dataset and annotation

The dataset used for training and validation of CNN models was composed of 1730 (80%) and 433 (20%) intraoral camera images, respectively, which were achieved before December 31, 2021. For the blind test of trained CNN models, 519 intraoral camera images were collected after January 1, 2022, which were used for index tests between the models and reference standard. Therefore, images included as test datasets were not used as training or validation datasets. In the training and validation datasets, 988 (57.2%) and 248 (57.2%) carious images were included, respectively. However, in the test dataset, 369 (71.0%) carious images were included. A total of 3685 carious lesions were included in the whole dataset (Table 1).

**Table 1:** Dataset composition.

Dataset	Total images	Dental Caries included images	Dental Caries not included images	Dental Caries lesion site included in dataset
Train set	1730(65%)	988	742	2599
Validation set	433(16%)	248	185	230
Test set	519(19%)	369	150	856
Total	2682(100%)	1605	1077	3685

As a reference standard, one board-certificated dentist specializing in government-led oral examination annotated dataset images about dental caries presence or not according to a clinical chart containing information on dental caries, surface, and severity, which were examined at the dental clinic. Also, box boundary was drawn for each carious lesion by one dentist based on the photographs in addition to clinical chart information using a personal computer according to the International Caries Detection and Assessment System (ICDAS) [42, 43]. If there were separated carious lesions in one tooth, each bounding box was independently created. Only ICDAS 4–6 codes were annotated as dental caries cases (ICDAS Code 4: An underlying dark shadow from the dentin with or without localized enamel breakdown, Code 5: Distinct cavity with a visible dentin, Code 6: Extensive distinct cavity with a visible dentin), which may be closely related to the necessity of dental treatment clinically [43, 44].

### 2.3 Deep learning algorithm application

**Fig 1:**
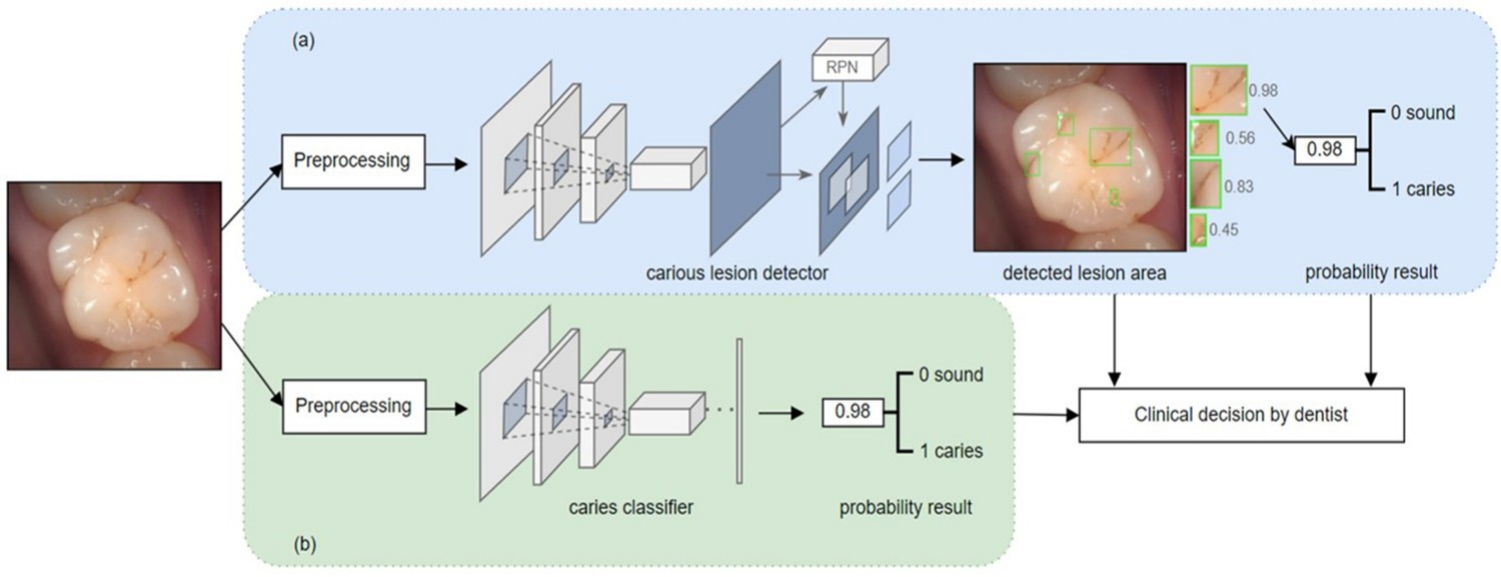
Block diagram of the experimental process for dental caries classification with (a) the object detection-based method and (b) the end-to-end image classification method.

In this study, two different approaches were used for dental caries classification: (1) end-to-end image classification and (2) object detection-based image classification. The first method used the ResNet-50 [7], Inception-v3 [45], and Inception-ResNet-v2 [46] models, which were selected owing to their validated performance in various image classification tasks. ResNet-50 uses skip connections to overcome the vanishing gradient problem for deep training crucial in dental imagery, effectively learning from residual data to detect subtle dental caries signs. Inception-v3, with parallel convolutions of different sizes within its modules, excels at identifying various stages and sizes of dental caries, with architectural advancements like factorized convolutions reducing computational costs while preserving performance. Inception-ResNet-v2 combines Inception’s and ResNet’s strengths, enhancing training speed and accuracy in dental caries detection by efficiently processing complex visual information in dental caries images. To apply these models to dental caries classification, they were fine-tuned using pre-trained models from the ImageNet dataset [47]. During the fine-tuning process, the dataset was preprocessed by resizing the images to a standard size and normalizing the pixel values. Various image augmentation techniques, including translation, rotation, brightness adjustment, and contrast adjustment, were employed. The last layer of each model was replaced with a new fully connected layer, followed by a softmax activation function for dental caries classification. Furthermore, the weights of the new layer were randomly initialized, and the pre-trained weights were loaded into the initialized model. All the weights of the model were then trained using the dental dataset with the Adam W optimizer [48], minimizing the cross-entropy loss function. The training dataset was divided into training and validation sets to monitor the performance of the model and prevent overfitting. Finally, a fine-tuned model was evaluated with test data. Fig 1(B) illustrates the first method. For the object detection-based method, the Faster R-CNN model with a ResNet-50 backbone was used. Faster R-CNN stands out in the field of computer vision by efficiently combining region proposals with deep learning for object detection. This model was selected owing to its efficacy in detecting objects within images and determining their positions [6]. The model was pre-trained on the COCO 2017 dataset [49], and the weights were fine-tuned using our dataset. During training using the object detection method, the dataset contained annotated bounding boxes indicating the instances of dental caries. In the same manner in the end-to-end image classification, the dataset underwent preprocessing, including image resizing, pixel value normalization, and image augmentation. The feature pyramid network was used to extract features at various scales [50]. Fig 1(A) illustrates the second method.

**Fig 2:**
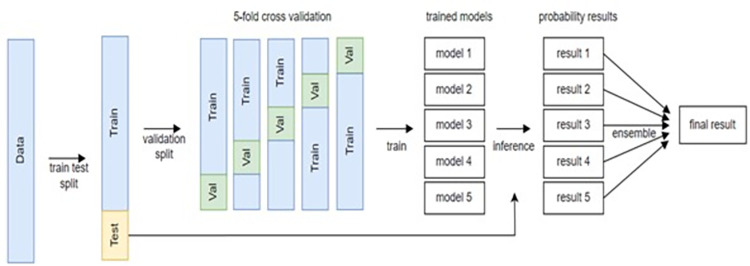
Data flowchart with ensemble inference for dental caries classification.

After conducting the experiments with the end-to-end image classification models (ResNet-50, Inception-v3, and Inception-ResNet-v2) and the object detection-based model (Faster R-CNN), additional experiments were conducted to apply an ensemble technique. As presented in Fig 2, the ensemble technique involved averaging of the resulting probabilities from the fivefold cross-validation results for each model. The ensemble technique was applied to the end-to-end image classification and object detection-based models. The following hyperparameters were used in CNN models: base learning rate: 0.001; batch size for classification/detection-based models: 32 / 4 (as possible as memory allowed); weight decay: 0.01; resize: (384, 384), flip probability: 0.5, random brightness contrast probability: 0.2, softnms sigma/threshold for detection-based model: 0.5 / 0.05.

### 2.4 Statistical analysis

As performance metrics for dental caries classification, sensitivity, specificity, precision, accuracy, F1 score, average precision (AP), and area under the receiver operating characteristic curve (AUROC) were measured. Sensitivity measures the proportion of actual positive instances correctly identified by the model, whereas specificity measures the proportion of negative ones. Precision quantifies the accuracy of positive predictions, and accuracy provides an overall assessment of the correctness of the model. The F1 score combines precision and recall into a single metric. Because sensitivity, specificity, precision, accuracy, and F1 score vary according to the threshold, Youden’s J statistic method was employed to find an appropriate threshold. Youden’s J statistic combines sensitivity and specificity to determine the optimal threshold for classification, striking a balance between true positives (TPs) and true negatives (TNs) [51]. AUROC evaluates the performance of the model across various threshold values by measuring the area under the ROC curve. AP is another metric that measures the overall precision at different recall levels, which was calculated by computing the precision at various recall thresholds and then averaging them. AUROC and AP are more important as they are less affected by the threshold than the other metrics [52]. For the evaluation of the model, we employed the Python programming language (Python; Python Software Foundation, Beaverton, OR, USA) and the scikit-learn library for measuring performance indicators such as accuracy, precision, recall, and F1 score.

## 3. Results

Using a test dataset consisting of 150 healthy tooth images and 369 carious tooth images, including 856 carious lesion images, according to ensemble application, the evaluation results of the classification models are presented in Table 2. All the models exhibited improved performance after applying ensemble at all the indices in which there were increases of 3.8%, 5.4%, and 3.5% for accuracy and 5.1%, 2.8%, and 2.8% for AUROC in ResNet-50, Inception-v3, and Inception-ResNet-v2, respectively. Among the three models, Inception-ResNet-v2 had the best performance indices: accuracy of 87.1%, specificity of 89.3%, precision of 95.2%, AUROC of 93.8%, and AP of 97.3%.

**Table 2:** Evaluation result s of end-to-end classification for dental caries images according to ensemble application.

Evaluation metrics	ResNet-50		Inception v3		Inception-ResNet-v2	
	Ensemble application of CNN model					
	No	Yes	No	Yes	No	Yes
True positive case	288	305	298	327	311	318
False positive case	22	19	22	23	27	16
False negative case	81	64	71	42	58	51
True negative case	128	131	128	127	123	134
Accuracy (95% CI)	0.80	0.84	0.82	0.86	0.84	0.87
	(0.77, 0.84)	(0.81, 0.87)	(0.79, 0.85)	(0.85, 0.90)	(0.80, 0.87)	(0.84, 0.90)
Sensitivity (95% CI)	0.78	0.83	0.81	0.89	0.84	0.86
	(0.74, 0.82)	(0.79, 0.86)	(0.77, 0.85)	(0.86, 0.92)	(0.81, 0.88)	(0.83, 0.89)
Specificity (95% CI)	0.85	0.87	0.85	0.85	0.82	0.89
	(0.79, 0.91)	(0.82, 0.93)	(0.79, 0.91)	(0.79, 0.90)	(0.76, 0.88)	(0.84, 0.94)
Precision (95% CI)	0.93	0.94	0.93	0.93	0.92	0.95
	(0.90, 0.96)	(0.91, 0.97)	(0.90, 0.96)	(0.91, 0.96)	(0.89, 0.95)	(0.93, 0.97)
F1 score (95% CI)	0.85	0.88	0.87	0.91	0.88	0.91
	(0.82, 0.88)	(0.86, 0.90)	(0.84, 0.89)	(0.89, 0.93)	(0.85, 0.90)	(0.88, 0.93)
AUROC*	0.87	0.92	0.90	0.93	0.91	0.94
(95% CI)	(0.84, 0.90)	(0.86, 0.94)	(0.87, 0.93)	(0.90, 0.95)	(0.88, 0.94)	(0.92, 0.96)
AP**	0.94	0.97	0.95	0.96	0.96	0.97
(95% CI)	(0.92, 0.96)	(0.95, 0.98)	(0.94, 0.97)	(0.95, 0.98)	(0.95, 0.97)	(0.96, 0.98)

* Area Under [the receiver operating characteristic (ROC)] Curve

** Average precision

Based on the localization of dental caries lesion, image classification was performed; the evaluation metrics are presented according to ensemble application in Table 3. Without ensemble application, the AUROC was 88.6% and AP 94.4%, which increased to 91.2% and 95.9% after ensemble, respectively. Furthermore, specificity and precision increased to 88.7% and 94.5%, respectively.

**Table 3:** Evaluation results of classification for dental caries image based on the localization results according to the application of ensemble technique.

Evaluation metrics	Classification for dental caries image using Faster R-CNN*	
	Ensemble application of CNN model	
	No	Yes
True positive case	313	292
False positive case	33	17
False negative case	56	77
True negative case	117	133
Accuracy (95% CI)	0.83 (0.79, 0.86)	0.82 (0.78, 0.85)
Sensitivity (95% CI)	0.85 (0.81, 0.88)	0.79 (0.75, 0.84)
Specificity (95% CI)	0.78 (0.71, 0.84)	0.89 (0.84, 0.83)
Precision (95% CI)	0.91 (0.87, 0.94)	0.95 (0.92, 0.97)
F1 score (95% CI)	0.88 (0.85, 0.90)	0.86 (0.83, 0.89)
AUROC** (95% CI)	0.89 (0.85, 0.92)	0.91 (0.89, 0.94)
AP*** (95% CI)	0.94 (0.92, 0.96)	0.96 (0.94, 0.98)

* Region-based Convolutional Neural Network

** Area Under [the receiver operating characteristic (ROC)] Curve

*** Average precision

**Fig 3:**
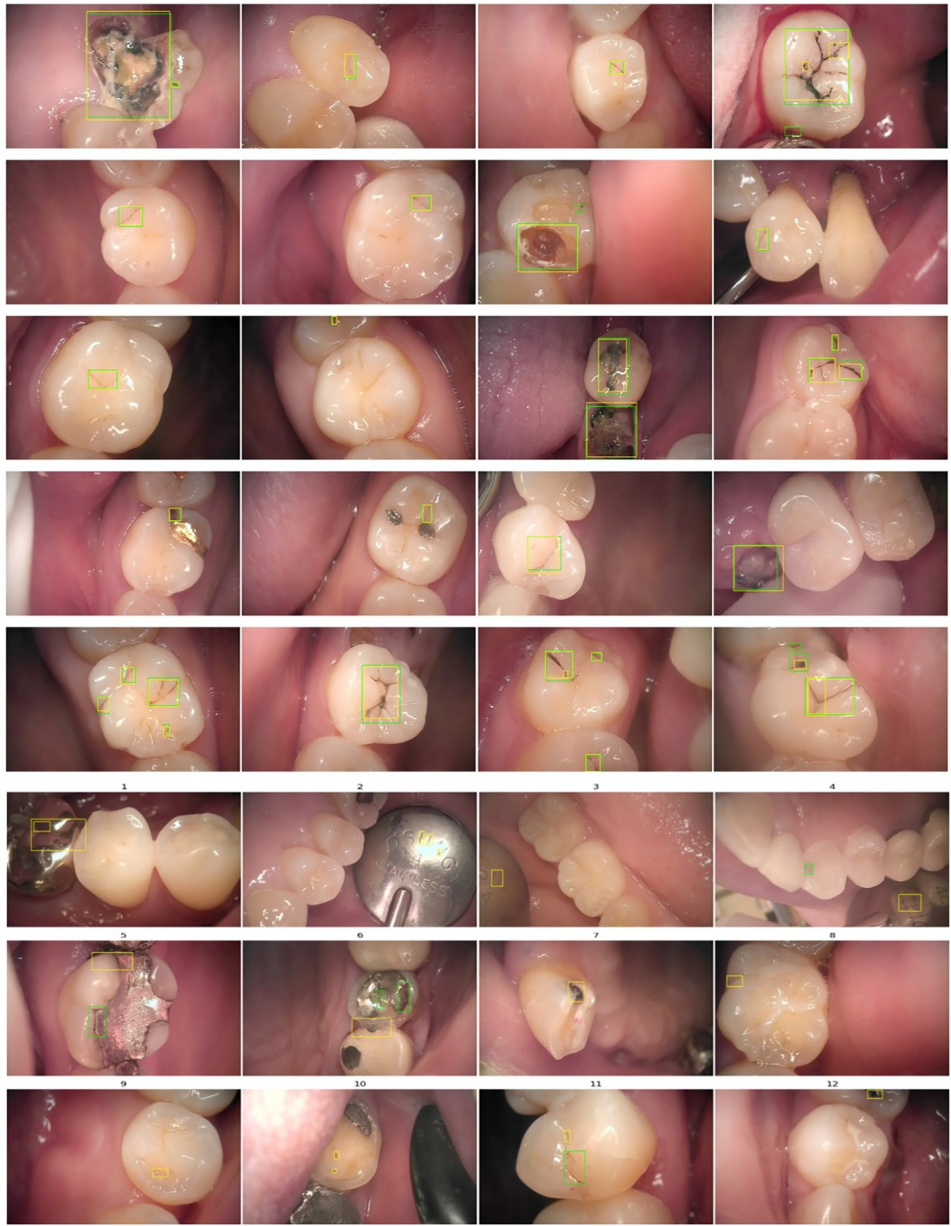
Qualitative results for carious lesion localization (green: Ground truth, yellow: Predicted). (a) Ture positive cases, (b) False positive & False negative cases.

The qualitative results of the localization of carious lesions are presented in Fig 3. In Fig 3(A), accurate results are shown, demonstrating a close alignment between the predicted dental caries locations and the ground truth. Dentists can use this information on predicted dental caries locations as medical evidence for the classification results. On the other hand, Fig 3(B) presents the cases where the predicted results do not align with the ground truth. These discrepancies can be attributed to the similarity in texture between the dental mirror (cases 2 to 4) and various types of implants (cases 1, 5, and 6) with dental caries. Consequently, these factors may lead to erroneous influences on the classification results, as illustrated in Fig 3(B).

## 4. Discussion

The ensemble technique application to the DL models consistently improved diagnostic performance in dental caries detection tasks in this study. As a result, the AUROCs of the DL models showed an increase of 2.6% to 5.1% after the ensemble application. Also, the increase in APs was 1.0% to 2.6% among the ensemble-applied DL models. Aside from these, most performance indices including Accuracy, Sensitivity, Specificity, Precision, and F1 score were improved in the ensemble-applied DL models except the Specificity in Inception v3, and Accuracy, Sensitivity, and F1 score in Faster R-CNN. The Ensemble technique combines multiple models to create a more robust and accurate system, which has been proven to be advantageous in enhancing overall performance [32–34]. By leveraging the strengths of each model and mitigating their weaknesses, ensembles achieve better performance results, which was confirmed in dental caries detection-DL models using intraoral photographic images in this study.

The intraoral camera photograph has several advantages in caries detection compared to traditional visual examination of subjects. Magnified vision and archiving of digital images might contribute to efficient early caries monitoring, higher sensitivity for caries detection, blinding of examiners in the comparative study, and remote dental examination for an epidemiological survey [53, 54]. Timely dental intervention according to early caries lesion monitoring could contribute to oral health by preserving tooth substance [3–5]. Therefore, high-performance DL models using intraoral camera images might contribute to patient oral health in various ways.

In the case of the end-to-end classification task, a consistent increase in TP cases and a decrease in false negative (FN) cases of all the ensemble models indicate that ensemble techniques are advantageous in correctly identifying carious images. In the case of noncarious images, two of all three models demonstrated better performance, an increase in TN cases, and a decrease in false positive cases, respectively. As a result, the improvement of most evaluation metrics with the application of the ensemble technique indicates its reliability and efficacy in accurately identifying dental caries from intraoral images. For example, the ensemble model of Inception-ResNet-v2 had the highest specificity (89.3%), precision (95.2%), AUROC (93.8%), and AP (97.3%), suggesting its proficiency in detecting dental caries images. On the other hand, Inception v3 exhibited high values for accuracy (87.5%), sensitivity (88.6%), and F1 score (91.0%). Its high sensitivity indicates a reduced risk of missing positive cases, which is crucial for effective diagnosis and timely treatment of dental caries. ResNet-50 demonstrated a competitive performance, which was slightly lagging behind. In conclusion, these models demonstrated their potential in dental caries detection and hold promise for further improvements with optimized training and fine-tuning.

Similarly, improved performance was observed with the application of ensemble techniques during the object detection-based classification task. Object detection, as a computer vision technique, provided valuable insights into the localization and identification of target objects. We supposed that localization of dental caries lesions enabled better understanding and explanation regarding the classification results of each image, which are essential for gaining trust and acceptance in clinical applications. In this study, based on this idea, classification was performed in such a way that if even one carious box predicted by the Faster R-CNN, exists, classification proceeds. These classification results indicated a favorable performance, showing an AUROC of 91.2% and an AP of 95.9%, which meant increments of 1.5% and 2.6% respectively after ensemble application. Compared with the end-to-end classification, the object detection-based classification had the most FN cases, which was 77, which implies a decision tendency toward non-carious images.

There were several strengths in this study. First, we confirmed the improved overall performance by ensemble application using various deep learning models (ResNet-50, Inception-v3, Inception-ResNet-v2, and Faster R-CNN). Second, Inception-ResNet-v2 showed the highest performances (i.e. 97.3% of AP, 93.8% of AUROC) after ensemble application, which was relatively favorable for clinical field use. Third, for the explainability of the DL model, object detection-based classification task was tried and showed favorable performance, which was first as far as our present knowledge goes.

However, detecting dental caries of AI models using intraoral photographs could not replace visual clinical examination because there were various dental caries types according to their severity, activity, and location such as interproximal surface, which may need the experience of clinicians or x-ray [55]. Nevertheless, the role of AI in dental caries detection appears promising considering its accessibility, convenience, and cost-benefit ratio along with improving accuracy in the future [56].

However, despite the promising performance of these ensemble models, there are still some challenges and areas for improvement. The first, the exact sample size for the test dataset was not calculated. However, based on the previous studies [12], assuming the accuracy of 0.8 and 0.75 of the CNN model and dentists with a standard deviation of 0.4, a study powered at 1-beta of 0.8 with alpha of 0.05 needed 442 teeth for the test dataset. Considering at least one or more teeth were included in each image of our dataset, the sample size of the test dataset (n = 519 images) could be regarded to be sufficient. The second, proximal-surface caries undetectable by visual examination was not regarded as dental caries during dataset annotation for better performance of the CNN model. Therefore, considering X-rays is essentially required for the diagnosis of proximal surfaces, our model could not replace the clinical visual examination. The third, only ICDAS codes 4–6 were annotated as dental caries considering the WHO caries assessment system, which had been designed for the reliability in epidemiological surveys. Given the convenience and cost-effectiveness of a camera image-based AI model, it might be useful in a community-based oral examination. Therefore, we used ICDAS code 4 as a cut-off point for caries annotation according to one recent study about the comparability of two systems i.e., WHO and ICDAS [57]. However, considering that ICDAS 1–3 codes were not annotated as caries in this study, our AI models might have limitations on the detection of early caries lesions. The fourth, the reference standard examiner made annotations according to caries lesion diagnosis based on the photographic images and clinical charts instead of direct examination on the patient without a report about the reproducibility of diagnosis. Therefore, this might create a measurement error and affect the accuracy of the results. Lastly, the dataset of the study and test scenarios might not fully represent the diverse cases encountered in clinical practice, leading to potential biases and limited generalization. To ensure the efficacy and robustness of the models in real-world applications, validation on larger and more diverse datasets is necessary. Furthermore, future research could explore the integration of additional data sources, such as patient records and medical imaging, to enhance the diagnostic capabilities of the model.

In conclusion, the ensemble applied-DL models consistently showed better-improved performances in dental caries detection using the traditional classification way, an end-to-end method, as well as an object (i.e., caries-lesion) based way which was designed for better explainability among dental clinicians. These results suggested ensemble application as a useful way to increase diagnostic performance in the DL models using intraoral camera images for dental caries detection with explainability.
